# Linking heat and electricity supply for domestic users: an example of power-to-gas integration in a building

**DOI:** 10.1039/d2ra00951j

**Published:** 2022-04-04

**Authors:** Emanuele Moioli

**Affiliations:** Thermochemical Processes Group, Energy and Environment Division, Paul Scherrer Institute Forschungstrasse 111 5232 Villigen Switzerland emanuele.moioli@psi.ch

## Abstract

A novel power-to-X system, coupling electricity and gas grid in a building, is presented. This system operates a retrofit of the existing photovoltaic system, consuming the electricity overproduction in the local synthesis of methane instead of injecting it into the electricity grid. Methane can be stored in the gas grid and used in winter in the existing gas burners, providing the required heat to keep the building at a comfortable temperature. Additionally, the methanation system provides waste heat that is used to warm up the sanitary water, eliminating the need for an electric boiler. The system, fed by 800 m^2^ of solar panels, was optimized according to the weather conditions and the dimensions of the main pieces of equipment were determined. This allows the production of *ca.* 17 MW h of methane for seasonal storage. By retrofitting the building with the power-to-X unit, the energetic independence of the house is maximized, thanks to the synchronous production of electricity, gas, and heat, including energy storage. Therefore, the profitability of the photovoltaic system is ensured independently from the electricity feed-in tariffs. The system performance was evaluated in a case study in the north of Switzerland. When considering the purchase of renewable natural gas (*i.e.*, from biogas), it was calculated that the system would be profitable for an electricity price below 0.05 € per kW h.

## Abstract 2

A power-to-gas system is integrated in a building to store the excess self-produced solar energy and reduce the heating expenses. This is possible by recovering waste heat from the reaction and using the gas grid as energy storage infrastructure.

## Graphical abstract

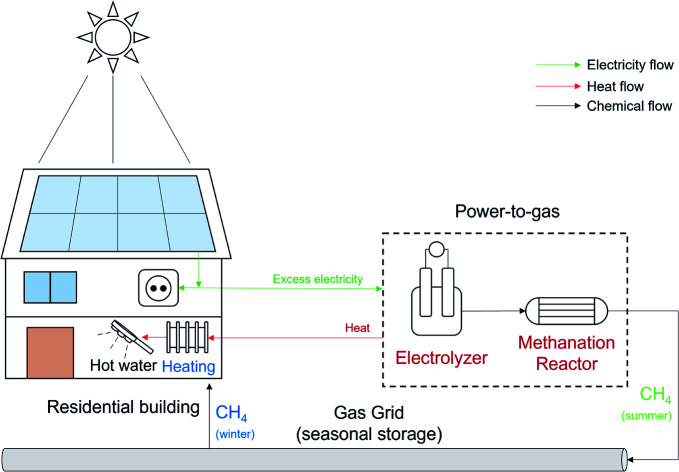

## 1. Introduction

The transition to clean energy in residential buildings must also ensure the supply of sustainable heat throughout the year. However, while several incentives are currently available for the installation of renewable energy harvesting devices in buildings (*e.g.*, solar panels on rooftops),^1^ initiatives addressing the self-production of heat are rare and mainly exploited through the installation of solar collectors in regions with large solar irradiation.^2^ The solar energy availability as well as the electricity and heat demand are often subject to temporal phase shifts, with the former being abundant in summer and during the day and the latter being required in winter and during the night. The combined action of all these phenomena causes an important mismatch between energy production and consumption, which can lead to problems in the technical and economic operation of the electricity grid.^3^ This may lead to two concurrent phenomena: excess electricity in summer, which could cause a drop in the energy price, and a deficit of energy in winter, which could lead to an increase in the price of energy. Additionally, the possible introduction of important carbon taxes may cause an important increase in the cost of standard gas-based heating systems.^4^

For all these reasons, it is essential to design new energy systems that can prepare residential buildings to cope both with the risk of not being able to place the excess electricity on the market in the summer and with the danger of a substantial rise in the gas bill in the winter. In this sense, state-of-the-art solutions for efficient heating, such as the use heat pumps, may contribute to the growth of the problem, because they require electricity precisely at the moment of a possible shortage. Hence, in this context, the concept of power-to-gas (PtG) becomes interesting for the energy supply of buildings. In fact, one may imagine using the excess electricity available in the summer (which has a low value for the above-mentioned reasons) for the local production of synthetic natural gas (SNG), *e.g.*, *via* water electrolysis and CO_2_ methanation.^5,6^ SNG can be stored in the natural gas grid as a carbon-neutral substitute of the standard fossil methane and then bought back in winter for consumption in standard gas-fired heating systems.^7^ In this way, the return on investment for solar panels installation is guaranteed over time and the de-fossilization of the heating system can be achieved. Such micro-scale energy storage may also significantly reduce the share of energy curtailment, while also increasing the penetration of renewable sources in to the market.^8^ Additionally, an optimal strategy combining energy storage and redispatch with an appropriate geographical distribution can raise the flexibility of the renewable energy sector.^9^ Unfortunately, work in this area has so far mainly focused on the creation of energy production islands, typically of much larger dimensions than a single building.

In this work, it is shown how a small-scale PtG system that could enhance the energetic self-independence of a single building can be optimally designed. This paper is distinguishes from the available literature in terms of its specific focus on the single building scale. The energy and heat supplies in this case study were designed as a whole from the available solar panels and the integration with the existing heating system and sanitary water structure. The system was based on a coupled electrolyzer/CO_2_ methanation block with an electrical power input below 50 kW, whose operation was tested in previous works.^10,11^ The dimensions of the main apparatus were determined based on the measured conditions at the target location (*e.g.*, solar irradiation and temperature). This work reports on the possibility of connecting the heat and energy supply at a small-scale, by recovering the waste heat of a PtG system from the heating of sanitary water. Furthermore, this work shows how a cross-disciplinary methodology can be applied under different economic and geographic boundary conditions.

## 2. Methodology

### 2.1 Source of data

The atmospheric data for the case study of Brugg (Switzerland) were collected on the basis of field measurements and according to data from the Swiss Federal Office of Energy (SFOE) and from the Federal Office of Meteorology and Climatology (MeteoSwiss).^12^ The solar energy and temperature profiles for Sion and Lugano were obtained from the website renewable.ninja.^13^ Comparison of the latter and the former data collection methods for Brugg yielded very similar results. The energy consumption data were collected from the Swiss Federal Office of Energy,^14^ on a household basis. The electricity availability profiles were calculated on an hourly base. For the sake of intelligibility, the results were summed up on a weekly or monthly basis for the figures displayed in this paper. The hot water requirements were calculated on the basis of real data collected in the field. The requirements corresponded to the supply for the 64 households living in the studied building. Hot water was delivered by the system at 80 °C. The requirement corresponded to *ca.* 50 L of hot water per person per day. The ambient heating requirements were calculated according to the measured temperature profiles and considering the influence of heat losses by conduction/convection and air leakage. The total heat requirements were calculated using the following equation:

1 *Q*_tot_ = *Q*_cond_ + *Q*_al_

The heat losses by conduction/convection were calculated from:

2 *Q*_cond_ = ∑*U*_i_*A*_i_(*T*_in_ − *T*_out_)

where *U*_i_ is the heat-transfer coefficient for each external element of the building, *A*_i_ is the surface of the component, and *T*_in_ and *T*_out_ are the internal and external temperatures, respectively. The main constructive elements considered were the concrete walls and the double-glazed windows. The heat losses by air leakage were calculated as:

3 *Q*_al_ = *ρ*_i_*c*_p_*q*_v_(*T*_in_ − *T*_out_)

where *ρ*_i_ is the air density (1.2 kg m^−3^), *c*_p_ is the specific heat capacity of air (1 kJ kg^−1^ K^−1^), and *q*_v_ is the air leakage flow. The air leakage flow was calculated as:4
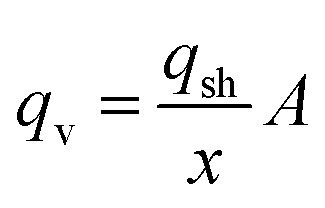
where *q*_sh_ is the air leakage number of the building shell (m^3^ h^−1^ m^−2^, here assumed as 4 m^3^ h^−1^ m^−2^), *x* is the storey factor (here assumed as 15, because of the large number of floors), and *A* is the external surface area of the building. The heating system was activated only when the mean daily temperature was below 12 °C. The internal temperature was fixed at 20 °C. The results of the calculations were compared with the yearly averaged real data available from the building, yielding similar results. The heat demand data were available on an hourly basis and therefore were directly comparable with the energy supply data.

### 2.2 System design

In the system, H_2_ was produced from water in a proton exchange membrane (PEM) electrolyzer. This unit was modeled with a black box approach according to the real data available.^15^ The efficiency of the operation (power to H_2_) was 65%, with the remaining electricity converted into waste heat. The waste heat was available at 50 °C. The dimensions of the electrolyzer were determined by optimization of the system cost, including for an intermediate battery for infra-day energy storage. The optimal point was a compromise between a big battery (small electrolyzer, thanks to the large peak shaving) and a big electrolyzer (need to operate when a large amount of electricity is instantaneously available). The optimization function is:5
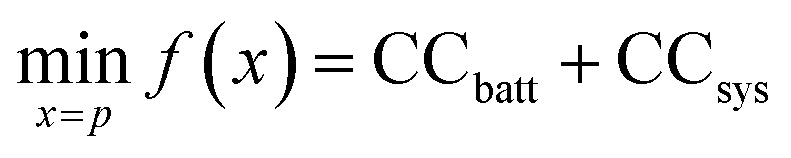

5a s.t.: *P*_in_ = *p* + *s*

5b s.t.: *V*_batt_ < 10 m^3^

5c s.t.: *s* > 0

where *p* is the productivity of the system (kW of synthetic natural gas), CC_batt_ is the capital cost of the battery, CC_sys_ is the capital cost of the reactive system, and *V*_batt_ is the battery volume. The equality constraint requires that the system is always equilibrated, which means the excess electricity (*P*_in_) is converted at any moment either into methane (*p*) or stored in the battery (*s*).

Once the dimensions of the electrolyzer were defined, the CO_2_ methanation reactor was designed to ensure the grid-compliant production of SNG at full load. The system design was performed on the basis of the canonical heat and mass balances for fixed-bed reactors,^16^ using Ni/Al_2_O_3_ as a catalyst, according to the kinetic model from Koschany *et al.*^17^ The reactor model used was a 1D heterogeneous model, which was found to satisfactorily represent the reactor operation in dynamic conditions.^18^ The detailed reactor model used is reported in the appendix. The target CO_2_ conversion was 95%, which could be achieved with the considered catalyst, when operating the reactor at 280 °C. To this purpose, the reactor was cooled with thermal oil. The reactor was controlled by adapting the thermal oil flow rate in such a way that the temperature in the hotspot does not pass 500 °C, considered as the upper limit to avoid catalyst sintering.^19^ In these conditions, the Ni catalysts show low deactivation, so that the catalyst replacement can be planned for every 2 years.^20^ H_2_ and CO_2_ were preheated to the inlet temperature by using a small fraction of the waste heat from the reactor, avoiding the need for external heating. The excess H_2_ in the product stream was removed through a membrane to reach the grid-injection requirement of 2 mol per mol%.^21^ The process pressure was set at 10 bar. The membrane used was a hollow fiber type (EVONIK SEPURAN® Green) and it was dimensioned on the basis of the results from the field experiments.^22^ The cleaned SNG was purified from water in a condenser and cooled to 50 °C using the cooling water coming from the electrolyzer. Prior to grid injection, the gas was dried by passing it over silica gel, to reach the water concentration requirements.^21^ The designed reactor showed a full load efficiency of about 75% (H_2_ to CH_4_), with the remaining energy recovered in the form of waste heat. The efficiency increased up to the maximum value of *ca.* 80% (ref. 23) with decreasing the gas load (due to the larger conversion). The reactor could quickly adapt to modifications in the gas load, ensuring the production of grid-compliant SNG in about 10 minutes from warm startup to 100% load.^10^

### 2.3 Techno-economic analysis

The dimensions of the equipment calculated in the system design phase were used for the determination of the system cost. The electrolyzer CAPEX was calculated on the basis of the electricity input, with a cost of 1200€ per kW_e_.^15^ The battery was a lithium-type, with a purchase cost of 100€ per kW h.^24^ The costs of the reactor and condenser were calculated based on the volume, according to the method of Ulrich and Vasudevan.^25^ The catalyst cost was 100€ per kg and the membrane cost was 1000€ per m^2^; the water desiccant (silica gel) cost 22€ per kg.^26^ The CO_2_ was delivered from a near wastewater treatment plant, equipped with a biogas-upgrading unit. Hence, the cost of CO_2_ was low at 20€ per tonne. Alternatively, in the future, the CO_2_ may be delivered from point capture and stored locally in liquid form. The expected lifetime of the system is 20 years and the interest rate is 6%. The yearly expenses for operation and maintenance were 5% for the reactor and 1.5% for the electrolyzer and the battery. The economic performance of the system was evaluated in terms of differences from the current conditions. This means that the current condition, with the excess electricity sold to the grid, was used as a baseline for the analysis. In this way, the investment cost for the solar panels was not considered, but it was kept as a sunk cost. Hence, the economic performance was the ratio between the income from the PtG system and the income from electricity feed-in, expressed as:6
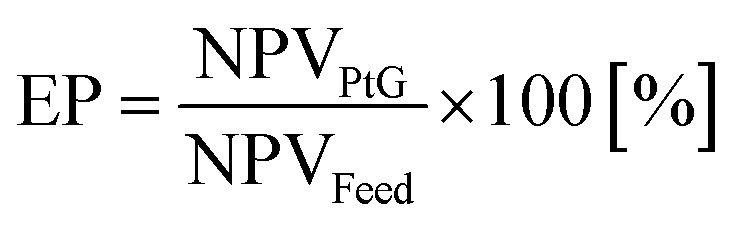
where EP is the economic performance and NPV is the net present value of the PtG and of the electricity feed-in. The NPV of the PtG can be expressed as:7
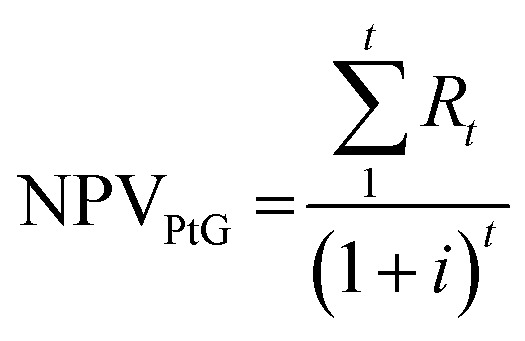
where *R*_*t*_ is the income at the time *t* and *i* is the interest rate. *R*_*t*_ is defined as:

8 *R*_*t*_ = CF_*t*_ − CAPEX_*t*_ − OPEX_*t*_

where CF_*t*_ is the cash flow at year *t*. CAPEX covers the capital expenditures of the PtG plant and OPEX covers the yearly expenditures related to operation and maintenance, spare parts replacement, and CO_2_ purchase. The NPV of the electricity feed-in is expressed as:

9 NPV_Feed_ = CF^e^_*t*_

where CF^e^_*t*_ is the cash flow related to the sale of the excess electricity.

## 3. Results and discussion

### 3.1 System design

**Fig. 1:**
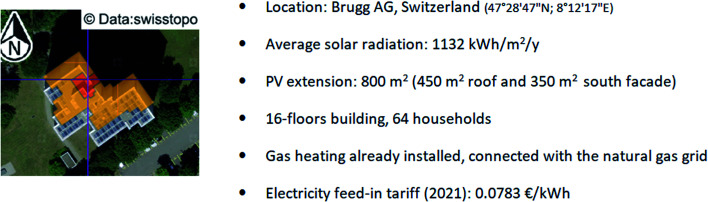
The main characteristics of the building considered in this study (red and yellow colors refer to two different areas with different expositions).

Fig. 1 summarizes the main characteristics of the target building retrofitted in this paper. The target building is located in Brugg, Switzerland. It is a large residential structure, composed of 16 floors, for 64 households. The building is already equipped with solar panels on the roof and on the façade for a total surface of 800 m^2^. Currently, sanitary water and ambient heating are provided by a centralized thermal station, operated with natural gas supplied from the local gas grid. Hence, the building has a direct connection with the gas distribution network. The local service provider (Industrielle Betriebe Brugg, IBB) currently purchases the electricity exceeding the building demand at a fixed rate of 0.0783€ per kW h. Hence, the building receives a contribution of about €15 000 per year from the excess electricity production.

**Fig. 2:**
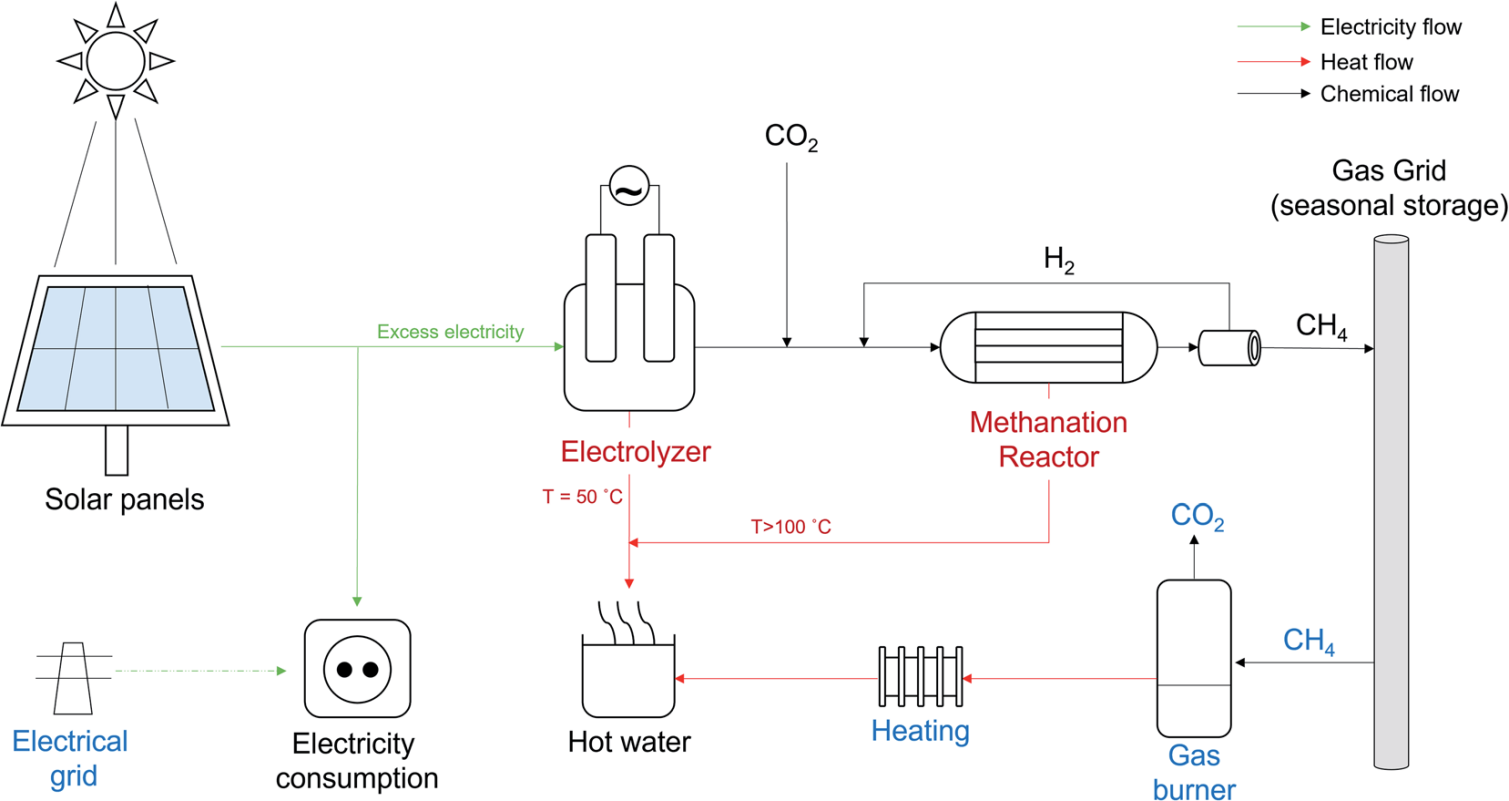
Schematic representation of the system designed. The equipment highlighted in red operates only when excess energy is available. The equipment highlighted in blue operates only when there is excess demand (heat and/or electricity).

The new system design is displayed in Fig. 2. Instead of being sold to the grid, the excess electricity (*i.e.*, what is not necessary to support the self-consumption of the building) is fed to the electrolyzer. A small battery (not shown in the figure) is installed with a peak-shaving function for ensuring the operation in case of prolonged bad meteorological conditions. The electrolyzer chosen was a proton exchange membrane type, to better adapt to the oscillations in the feed power while assuring a reasonable efficiency. The hydrogen produced is directly fed to the methanation reactor, without intermediate hydrogen storage, to limit the capital cost (CAPEX). The reactor operates with an highly efficient nickel-based catalyst, which can achieve high CO_2_ conversion.^11^ In order to reach the required methane purity (CH_4_ min = 96% vol/vol (ref. 27)), a small recycle membrane was installed.^28^ The reactor/membrane system can adapt well to the modifications in the flow rate, ensuring the production of grid-compliant synthetic natural gas.^22^ The entire system was equipped with non-return valves, to avoid the instauration of reverse flow conditions at the load change. The reactor is cooled with a thermal oil, warranting a good temperature control, essential to maintaining a high reactor performance.^29^ The electrolyzer is cooled with water, with an outlet temperature of about 50 °C. This water is then brought to the target temperature (*ca.* 80 °C) by removing the waste heat from the methanation reactor (cooling of the thermal oil and cooling of the product gas to condense water). The total amount of heat produced corresponds to *ca.* 48% of the total power input from electricity. The waste heat from this circuit is used to warm up the sanitary water for the building. The feasibility of this waste heat recovery system was already proven in the field.^30^ The methane product is directly injected in the gas grid, with a sell and buy contract (*i.e.*, the gas injected in summer is purchased back in summer without additional costs). This is possible thanks to the storage capacity of the gas grid, which allows a temporal shift between gas injection and consumption (contrary to the electricity grid). When the ambient temperature is below the comfort temperature (*i.e.*, 12 °C daily average), the heating system is activated (independently from the PtG system), consuming natural gas from the grid. In case the PtG system is not working, the heating system provides also the heat for the sanitary water.

The function of the PtG system is hence to valorize the excess electricity in the production of synthetic natural gas, making the building independent from oscillations in the electricity price. Furthermore, it allows the elimination of the need for natural gas consumption during summer, due to the need for sanitary water. Hence, the profitability of the system is enhanced by the utilization of the waste heat from the electrolyzer/reactor block. A connection to the electricity gas grid was necessary to ensure the supply in times with electricity deficit (*e.g.*, winter).

### 3.2 System performance

**Fig. 3:**
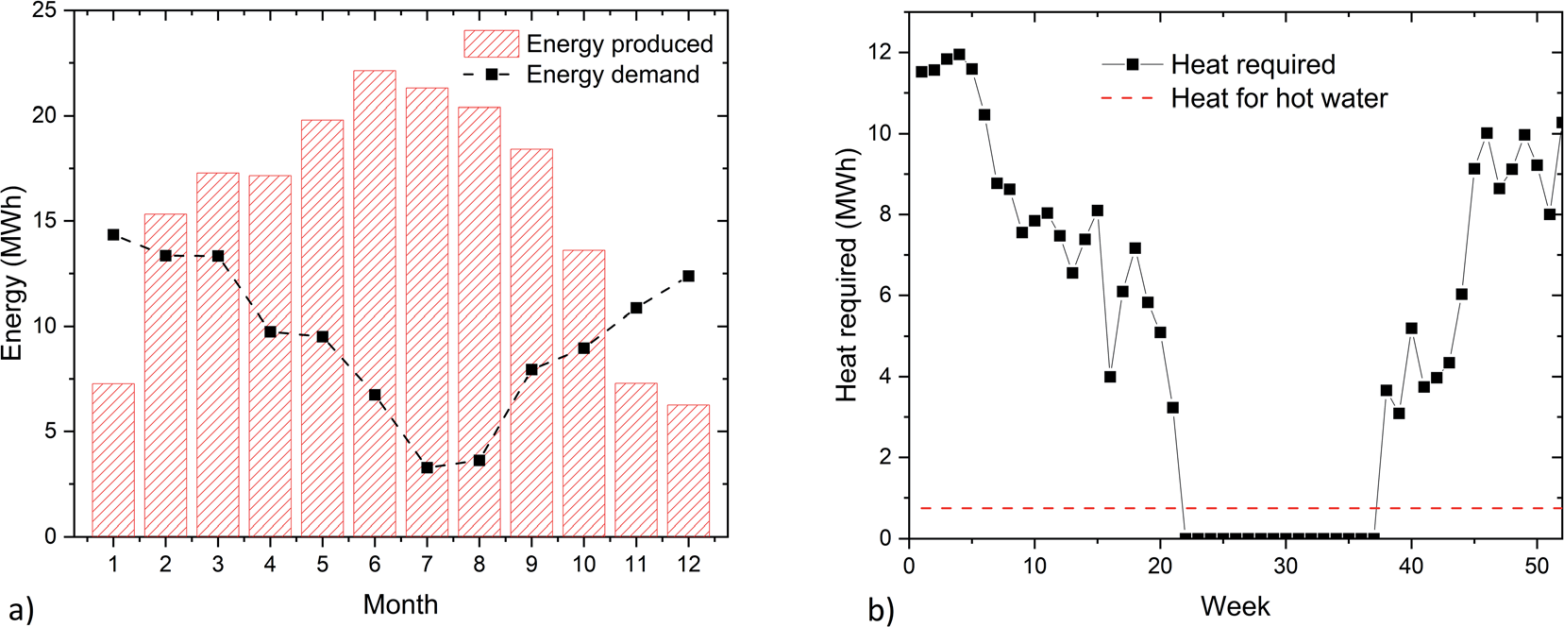
The main parameters required for the system design: (a) the energy availability from the solar panels and the electricity requirements of the building, (b) the heat required in the building in the form of ambient heat and hot sanitary water.

**Fig. 4:**
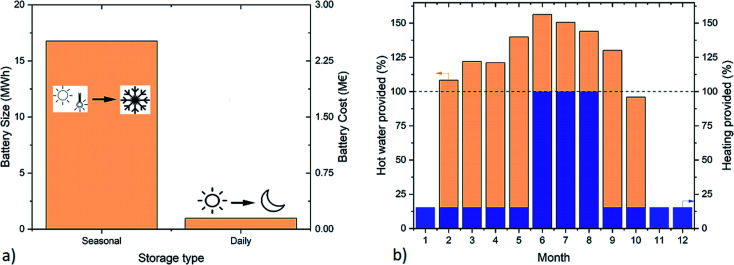
The main results of the system design: (a) dimensions of the required battery for seasonal and daily storage, (b) distribution of the produced heat, in the form of hot water and ambient heat.

The design of the system requires the analysis of the meteorological conditions expected at the selected location. Fig. 3 reports the main parameters needed for the system design. The seasonal variability in electricity supply between summer and winter is evident from Fig. 3a. The electricity production in the period November–January is particularly limited due to the low number of hours of sun irradiation and due to the tendency of fog formation. On the contrary, the electricity production in summer is large, thanks to the good irradiation and the geographical exposition of the building. The electricity consumption profile shows instead the opposite shape, with the maximum in winter, due to the larger need for lighting. This results in an important surplus of electricity in summer, which can be stored in the form of methane. Intermediate storage *via* a battery allows overcoming the day/night difference and ensures the supply in case of a prolonged lack of sunlight. This element was dimensioned with the aim to safeguard the operation of the system for at least 2 days in summer (to supply the demand from the building and the PtG system) or 5 days in spring (to supply the building plus the PtG system in standby). As shown in Fig. 4a, the size of the battery was approximately 0.5 MW h, which is an affordable investment, given the advantages achieved in the operation of the system. According to this assumption, the size of the electrolyzer was determined to be about 31 kW_e_. This corresponded to *ca.* 16 kW of waste heat available for sanitary hot water, sufficient to cover the demand from the building. The investment required to purchase a battery for seasonal storage (*i.e.*, storing electricity in summer to compensate for the energy deficit in winter) is otherwise excessive, at *ca.* €2.5 M, as shown in Fig. 4a. For this reason, it is more convenient to entirely store the excess electricity in methane and to purchase electricity in winter to compensate for the excess of demand. The heat demand of the building is shown in Fig. 3b, distinguishing between the heat requirements for ambient heating and for the hot water supply. The former shows large oscillations, linked to the ambient temperature, while the latter is almost constant during the year. Hence, the possibility of supplying the hot water *via* valorization of the PtG system was verified. The results of the calculations are reported in Fig. 4b. The system produces enough heat during 10 months per year (February to October). This means that during this period, the entire electricity surplus is used to produce H_2_ and consecutively methane. During the remaining months, the hot water must be produced by the heating system. However, these correspond to the fraction of the year when the heating system is constantly active, hence it can produce the required hot water with high efficiency (*e.g.*, with a condensation system).^31^ Interestingly, in this way, a dedicated burner for the hot water production could be removed, as it is not necessary in summer. Hence, the system makes the building completely carbon free for the electricity and heat supply in summer. In particular, the methane produced is sufficient to cover the heat demand in June, July, and August and an average of 15% of the requirements in the remaining months, as shown in Fig. 4b. This results in the supply of *ca.* 7300 Nm^3^ per year of methane from the PtG system, which corresponds to *ca.* 31% of the required ambient heat, reducing the global carbon footprint of the system by about 20% (Table 1).

**Table 1:** The main results of the simulations for the three locations considered

	Brugg	Sion	Lugano
Electrolyser size (kW)	30.73	33.00	31.85
Months PtG on	9	9	10
Excess electricity^a^ (MW h per year)	72	111	89
Methane production (Nm^3^ per year)	7293	8409	8237
Heat demand (w/o hot water) (MW h per year)	258	447	244
Self-sufficiency^b^ (%)	31.25	20.81	37.40
Economic performance^c^ (%)	17.76	20.96	21.14
Seasonal storage battery (kW h)	16761	3138	7170
Start storage	August	October	September
CO_2_ savings (%)	19.63	20.65	23.36

a Excess electricity = electricity produced − electricity consumed in the building.

b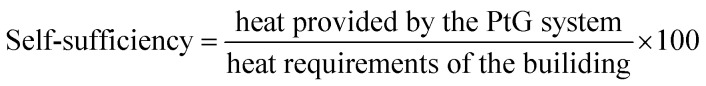.

c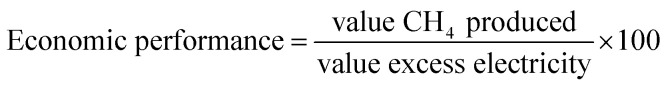.

**Fig. 5:**
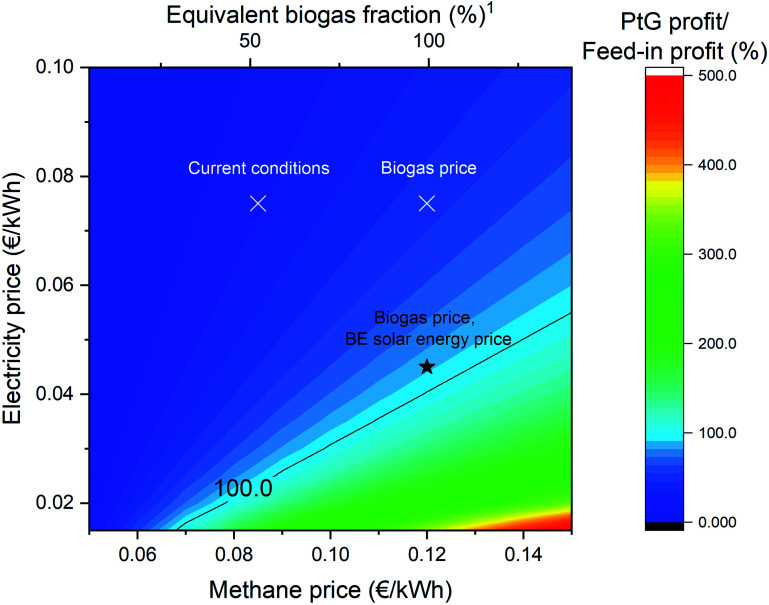
The calculated economic performance of the system, normalized to the income from electricity feed-in. The contour plot represent the ratio profit from PtG operation/profit from electricity feed-in. The results were calculated with varying the price of electricity (feed-in) and methane. The equality line (100%, *i.e.*, equal profit from the two systems) is highlighted. ^1^The biogas price was calculated according to ref. 34. Current conditions and biogas price refer to the prices applied in Switzerland in 2021.

The economic performance of the system was assessed in comparison with the current income guaranteed by the feed-in tariff (*i.e.*, determining the ratio between the savings on the methane bill and the revenue from selling the excess electricity). The PtG performance was calculated including the capital expenses for the purchase of the main equipment (divided over an expected lifetime of 20 years) and the operative expenditures linked to the consumables, operation, and maintenance. The results are reported in Fig. 5. Under the current conditions (methane price = 0.083€ per kW h), the economic performance of the PtG system is significantly lower than the electricity feed-in, with a profit ratio of approximately 18% (*i.e.*, the savings from not purchasing natural gas reach 18% of the possible profit from selling electricity). Even considering the current price of biomethane (0.12€ per kW h), the profitability is low, with a ratio of 50%. Hence, the system is profitable with the current feed-in tariffs. To profitably operate the system, the electricity price must be significantly lower. This would correspond to an electricity price of 0.025€ per kW h when considering the standard methane price and an electricity price of 0.045€ per kW h when considering the biomethane price. Hence, it was observed that the viability of the system is guaranteed only when the electricity price is low, namely in the renewable energy storage context. This confirms our initial assumptions that the system can operate if the energy storage is favorited by the economic conditions. However, it was also observed that, at the current biomethane price, the break-even electricity price corresponds to a value below the current production cost of solar energy (below 0.05€ per kW h (ref. 32)). Therefore, the elimination of the feed-in electricity incentives and the opening of the solar energy market could induce the development of these kinds of PtG systems, to ensure an adequate valorization of the produced electricity. Additionally, a decrease in the equipment cost (especially the electrolyzer) could have an important role in the increased applicability of this technology in the near future.^33^

### 3.3 Geographic sensitivity

**Fig. 6:**
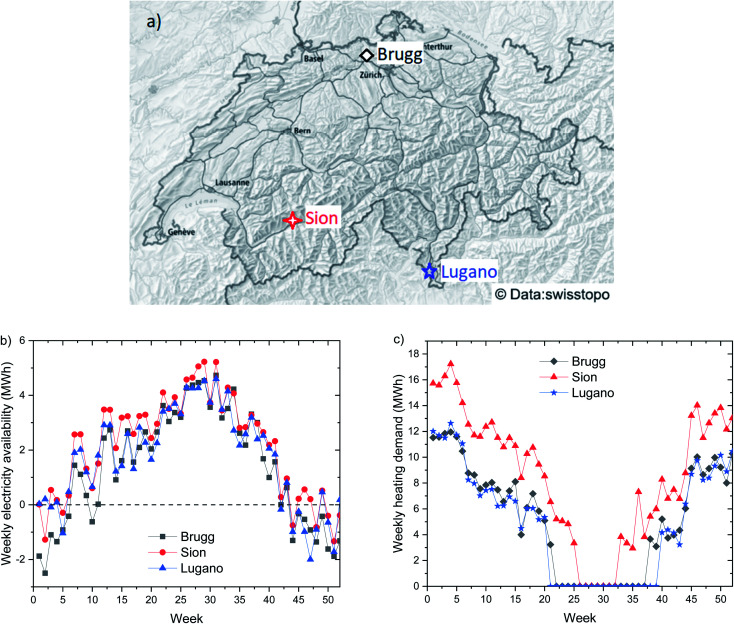
Input data for the geographic sensitivity analysis: (a) the geographical positioning of the locations selected for the sensitivity analysis, (b) the electricity availability profile over the year for the three locations (negative numbers indicate an excess demand), (c) the heating demand over the year for the three locations.

The results exposed in the previous section are valid for the specific case of the target building located in Brugg, Switzerland. To understand the effect of geography on the results, a geographic sensitivity analysis was performed. To this purpose, two further locations in Switzerland were selected: Sion and Lugano. The positioning of the three locations is shown in Fig. 6a. Brugg is in the north of Switzerland, in an area subject to fog in winter. As a result, electricity availability is low in November, December, and January, as shown in Fig. 6b (see also Fig. 3a). Sion is located in a valley in the Alps, characterized by a large availability of sunlight throughout the year. Hence, the energy availability is significantly larger than in Brugg (Fig. 6b). However, the average temperature in winter is significantly lower, thus requiring a larger amount of heat, as shown in Fig. 6c. Lugano is located on the south side of the Alps, and is therefore preserved from most of the cold air outbreaks from the north. Hence, the heat requirements are significantly lower, as visible in Fig. 6c. The solar energy availability in Lugano is instead intermediate between the two previous cases.

The results of the calculations are summarized in Table 1. The optimal size of the system was slightly larger in Sion (*ca.* 33 kW), due to the larger availability of solar energy, especially in summer. However, this did not increase the number of months of operation of the system, which corresponded to nine months for both Sion and Brugg. The operation window of the system was extended only in Lugano (10 months in total), thanks to the larger energy availability in winter. The larger size of the electrolyzer in Sion did not result in a higher self-sufficiency of the system (*ca.* 21% *vs. ca.* 31% in Brugg). This is due to the larger heat requirements of the building, resulting in a lower fraction of the needed methane that is produced by the PtG system. The self-sufficiency reached a maximum in Lugano, thanks to the best electricity availability/heat demand ratio (self-sufficiency of *ca.* 37%). The trend of self-sufficiency was not reflected in the economic performance of the system. In fact, the maximum of this indicator was found in Lugano, with 21.14% of the feed-in profit. The second best location was Sion, with a value of 20.96%, followed by Brugg with 17.76%. Hence, the profitability of the system was dependent both on the energy availability/demand ratio, but also on the degree of utilization of the system, which was lower in Brugg, due to the lower electricity availability in winter, spring, and autumn. This was also reflected in the size of the battery eventually required for the seasonal energy storage, which would be 16761 kW h for Brugg, 3138 kW h for Sion, and 7170 kW h for Lugano. Hence, it is demonstrated that the integration of a PtG system is a complex problem, requiring the optimization of contrasting objective functions (self-sufficiency, economic performance, carbon neutrality). The optimal solution strongly depends on the boundary conditions, including the geographic exposition, the shape of the building, and the consumption profiles.

## 4. Conclusions

In this work, a power-to-gas system was implemented in a building to connect the electricity production from solar panels and the heat supply in a gas-fired burner. The energy storage was enabled by the production of synthetic natural gas followed by direct injection in the gas grid. Additionally, the waste heat from the PtG system was used to warm up the sanitary hot water, producing significant natural gas savings. In this way, the building did not need to consume natural gas in summer and produces part of the gas required for winter. It was observed that the system considered in Brugg (Switzerland) could provide more than 30% of the yearly heat requirements of the building. It was observed that this value could vary significantly according to the solar energy availability and heat requirements of a specific geographic location. Nevertheless, in Switzerland, the variation band for this value in urban areas ranged between 20% and 40%. Despite the interesting effect in increasing the energetic self-sufficiency of the building, the economic profitability of the system was low compared to the current feed-in conditions. Hence, the development of these types of micro-scale PtG systems depends strongly on the pricing landscape for the electricity feed-in and the purchase cost of natural gas. However, if the produced SNG is considered renewable and hence awarded a high value (*ca.* 0.12€ per kW h), the required electricity price is in the current range of solar electricity break-even price (*ca.* 0.05€ per kW h). Therefore, the solution here proposed is feasible with the existing technology and can provide an important strategy for the reduction of the carbon footprint of buildings. In particular, the increase of the energetic independence of buildings is an essential step toward the development of cleaner cities. In this sense, the link between electricity production and heat supply is crucial and can be achieved with the strategy here presented. This increases the efficiency of the system, as it allows an appropriate utilization of the waste heat. The further evolution of the concept is linked to the modification of the feed-in regulations and toward the enhancement of the energetic independence of buildings, and could be supported by further decreases in the production cost of the components, *e.g.*, by production in series of the reactor-electrolyzer system. In the absence of these conditions, the micro-scale PtG may not become profitable in the near future and the focus of energy storage development might then only focus on the storage of solar energy from large harvesting facilities (providing energy at lower price), preferentially linked with large CO_2_ emitters (providing continuous carbon supply). This may be the case for buildings only at the district scale, with centralized energy storage facilities.

## Appendix

### Reactor model

The reactor was modeled with a dynamic 1D pseudo-homogeneous model with the catalyst effectiveness factor:A1
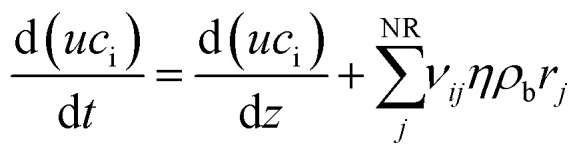
A2
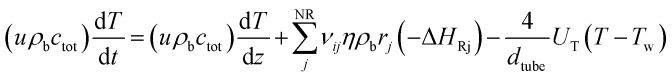


The WGS/RWGS reaction was modeled according to Xu and Froment's model.^35^ The temperature of the cooling fluid (*T*_e_) was calculated by balance in the water circuit:A3
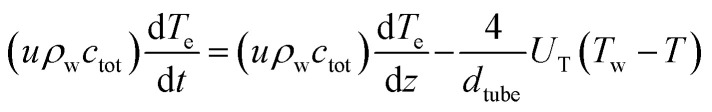


The catalyst effectiveness factor was calculated *via* the generalized Thiele modulus, calculating at each step the apparent reaction order:A4
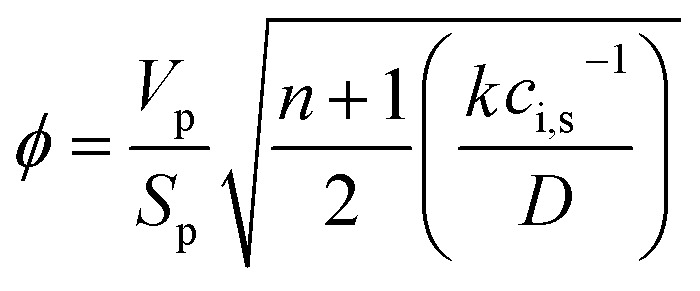
A5
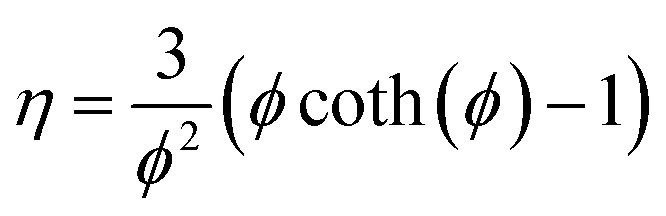


The heat-transfer coefficient was calculated as:A6
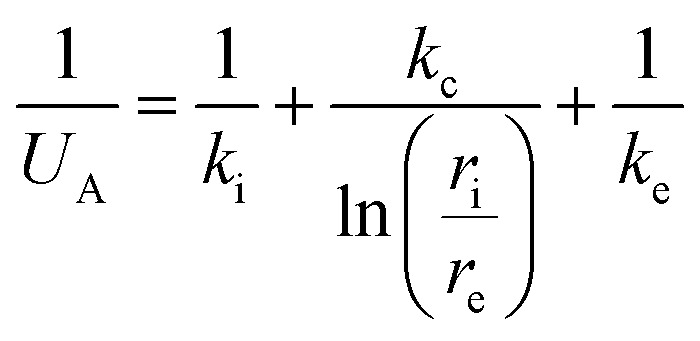


*k* was calculated considering a stagnant and a dynamic contribution:^36^A7
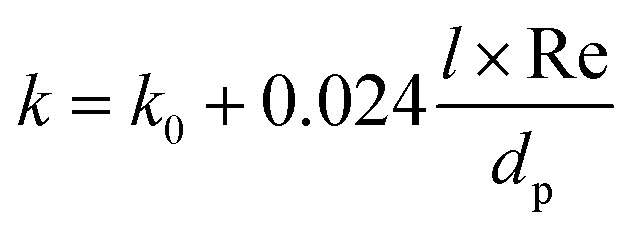


The superficial velocity of the gas was calculated with the continuity equation:A8
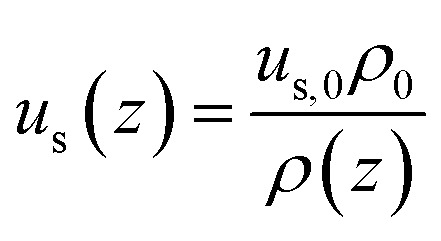


The equilibrium constant of the Sabatier reaction was calculated as:^17^A9
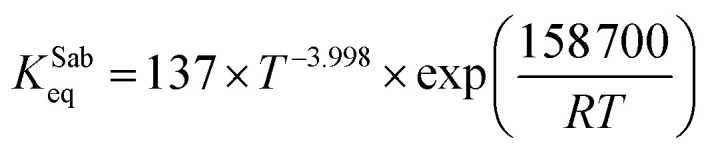


The equilibrium constant of the WGS reaction was calculated as:^37^A10
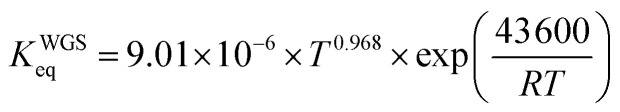


The boundary conditions were:

A11 *c*_i_ = *c*^0^_i_

A12 *T* = *T*^0^

A13 |^*x*=*L*^ = *T*^0^_w_*T*_W_

The reactor was 3 meters long, with a diameter of 0.01 m and a pellet size of 1.5 mm.

## List of symbols

*A*_i_
: Surface of the building (m^2^)
CAPEX: Capital expenditures (€)
CC_batt_: Capital cost of the battery (€)
CC_sys_: Capital cost of the reactive system (€)
CF_*t*_: Cash flow at the year *t* (€)
CF^e^_*t*_: Cash flow related to the sale of the excess electricity (€)
*c*_p_
: Specific heat capacity (kJ kg^−1^ K^−1^)
*d*_tube_
: Diameter of the reactor (m)
EP: Economic performance (€)
*i*
: Interest rate (−)
*k*
: Heat conductivity (W m^−2^ K^−1^)
*n*
: Reaction order (−)
NPV: Net present value (€)
OPEX: Operative expenditures (€)
PtG: Power-to-gas
*p*
: Productivity of the system (kW)
*P*_in_
: Excess electricity (kW)
*Q*_al_
: Air leakage heat losses (W)
*Q*_cond_
: Conductive heat losses (W)
*q*_sh_
: Air leakage number of the building shell (m^3^ h^−1^ m^−2^)
*Q*_tot_
: Total heat losses (W)
*q*_v_
: The air leakage flow (m^3^ s^−1^)
*r*_j_
: Reaction rate (mol m^−3^ s^−1^)
*R*
: Universal gas constant (J mol^−1^ K^−1^)
*R*_*t*_
: Income at year *t* (€)
*s*
: Battery power (kW)
*T*_in_
: Internal temperature (K)
*T*_out_
: External temperature (K)
*T*_w_
: Water temperature (K)
*U*_i_
: Heat-transfer coefficient (W m^−2^ K^−1^)
*x*
: Storey factor (−)
WGS: Water gas shift reaction
*ρ*_i_
: Density (kg m^−3^)
*ϕ*
: Thiele modulus (−)
Δ*H*_Rj_: Reaction enthalpy (kJ mol^−1^)
*ν*_*ij*_
: Stoichiometric coefficient (−)
*η*
: Catalyst effectiveness factor (−)

## Conflicts of interest

There are no conflicts to declare.

## Supplementary Material
